# Multimorbidity impacts cardiovascular disease risk following percutaneous coronary intervention: latent class analysis of the Melbourne Interventional Group (MIG) registry

**DOI:** 10.1186/s12872-023-03636-7

**Published:** 2024-01-23

**Authors:** Chau Le Bao Ho, Si Si, Angela Brennan, Tom Briffa, Dion Stub, Andrew Ajani, Christopher M Reid

**Affiliations:** 1 NHMRC Centre of Research Excellence in Cardiovascular Outcomes Improvement, Curtin University, Bentley, WA 6102 Australia; 2 Cardiovascular Research and Education in Therapeutics, School of Public Health and Preventive Medicine, Monash University, Melbourne, Australia; 3 School of Population and Global Health, the University of Western Australia, Perth, Australia

**Keywords:** Multimorbidity, Cardiovascular Disease, Latent class analysis

## Abstract

**Background:**

Multimorbidity is strongly associated with disability or functional decline, poor quality of life and high consumption of health care services. This study aimed (1) To identify patterns of multimorbidity among patients undergoing first recorded percutaneous coronary intervention (PCI); (2) To explore the association between the identified patterns of multimorbidity on length of hospital stay, 30-day and 12- month risk of major adverse cardiac and cerebrovascular events (MACCE) after PCI.

**Methods:**

A retrospective cohort study of the Melbourne Interventional Group (MIG) registry. This study included 14,025 participants who underwent their first PCI from 2005 to 2015 in Victoria, Australia. Based on a probabilistic modelling approach, Latent class analysis was adopted to classify clusters of people who shared similar combinations and magnitude of the comorbidity of interest. Logistic regression models were used to estimate odd ratios and 95% confidence interval (CI) for the 30-day and 12-month MACCE.

**Results:**

More than two-thirds of patients had multimorbidity, with the most prevalent conditions being hypertension (59%) and dyslipidaemia (60%). Four distinctive multimorbidity clusters were identified each with significant associations for higher risk of 30-day and 12-month MACCE. The cluster B had the highest risk of 30-day MACCE event that was characterised by a high prevalence of reduced estimated glomerular filtration rate (92%), hypertension (73%) and reduced ejection fraction (EF) (57%). The cluster C, characterised by a high prevalence of hypertension (94%), dyslipidaemia (88%), reduced eGFR (87%), diabetes (73%) and reduced EF (65%) had the highest risk of 12-month MACCE and highest length of hospital stay.

**Conclusion:**

Hypertension and dyslipidaemia are prevalent in at least four in ten patients undergoing coronary angioplasty. This study showed that clusters of patients with multimorbidity had significantly different risk of 30-day and 12-month MACCE after PCI. This suggests the necessity for treatment approaches that are more personalised and customised to enhance patient outcomes and the quality of care delivered to patients in various comorbidity clusters. These results should be validated in a prospective cohort and to evaluate the potential impacts of these clusters on the prevention of MACCE after PCI.

**Supplementary Information:**

The online version contains supplementary material available at 10.1186/s12872-023-03636-7.

## Background

More people are surviving acute coronary events largely attributed to advances in medical care and improved risk factor management. Consequently, more people are living with two or more chronic diseases; so called multimorbidity [1–5]. In Australia, nearly half of patients presenting at General Practices and one third of the general population report multimorbidity [6]. A recent study by Lai et al. 2019 observed a progressively increased rate of hospital admission in middle aged participants with multimorbidity as compared to those at older age [7]. Multimorbidity is strongly associated with disability or functional decline, poor quality of life and high consumption of health care services [2, 8, 9].

Growing epidemiological research examining the prevalence of multimorbidity has mostly focused on investigating the number of co-existing conditions or there dyad/triad combinations [10, 11]. In a UK study, three multimorbidity phenotypic clusters (low, medium and high levels of multimorbidity) was identified among patients with acute myocardial infarction (AMI) and found a dose-response relationship between the extent of multimorbidity and long-term survival [12]. This study used both cluster and latent class analyses that enable the distinction of co-morbid conditions that occur by chance from those that are statistically more likely to co-exist due to pathophysiological causes and/or socioeconomic or behavioural factors [13, 14]. However, the UK study restricted to AMI patients with single endpoint of all-cause mortality. Recently, patterns of multimorbidity was considered given the potential overlapping pathophysiological pathways [15] and thereby inform the allocation of health services [16, 17]. A study by Islam et al. further reported that it is not the number, but the patterns of multimorbidity that predict health service use [18]. Using a similar latent class analysis (LCA) method, this current study aimed to identify (1) patterns of multimorbidity among patients undergoing first recorded percutaneous coronary investigation (PCI) (both emergency and elective procedures); (2) the association between the identified patterns of multimorbidity on length of hospital stay, 30-day and 12- month risk of major adverse cardiac and cerebrovascular events (MACCE) after PCI.

## Methods

### Study design, settings and population

We conducted a retrospective cohort study of the Melbourne Interventional Group (MIG) registry [19]. The MIG registry included 14,025 participants who underwent their first PCI from 2005 to 2015 at six tertiary referral hospitals in Victoria, Australia. The registry recorded data on patients’ demographic characteristics, medical conditions at admission; PCI procedure; admission presentation, length of hospital stay, 30-day and 12-month MACCE at post procedure date. In this study, MACCE was defined deaths, non-fatal myocardial infarction, stroke and target vessel revascularisation. Follow-up information at 30-day and 12-month after PCI was collected in a standardised case report forms via telephone interview or record review by site nurse coordinators.

### Statistical analyses

Based on a probabilistic modelling approach, LCA was adopted to classify clusters of people who shared similar combinations and magnitude of the comorbidity of interest. The comorbidity of interest included: (1) hypertension, (2) dyslipidaemia, (3) diabetes mellitus, (4) chronic lung disease (CLD), (5) obstructive sleep apnoea (OSA), (6) reduced ejection fraction (EF < 45%), (7) peripheral vascular disease (PVD), (8) cerebrovascular disease, and (9) reduced estimated glomerular filtration rate (eGFR < 60 ml/min/1.73m2). Each morbidity in the model is classified into one of the latent classes according to their maximum likelihood class membership [20]. LCA incorporates standard procedures to handle missing values before assigning estimated probabilities of an individual belonging to distinct clusters [20]. Model selection was based on model fit parameters including likelihood-ratio G2 statistic, Akaike Information Criterion (AIC), Bayesian Information Criterion (BIC) and entropy.

Descriptive analyses were used to summarise the characteristics of patients according to comorbidity clusters identified by LCA. Differences between the comorbidity clusters groups for baseline characteristics were tested by t-tests for continuous variables and Chi-squared tests for categorical variables. Logistic regression models were used to estimate odd ratios and 95% confidence interval (CI) for the 30-day and 12-month MACCE. Both logistic and negative binomial regression models were performed for the association between comorbidity clusters and length of hospital stay. The models were adjusted for age, gender and other risk factors. The statistical significance was determined with a two-sided p-value below 0.05. Data analyses were performed using STATA (version 14).

## Results

In Table 1, more than half of the included patients were males (74%) and had a mean age of 63 ± 12 years. In the PCI cohort, hypertension and dyslipidaemia were the most prevalent conditions (59%); followed by diabetes, reduced EF and reduced eGFR in approximately 20% and the remaining conditions accounted for proportions < 10%. Indications for PCI included 78% of patients presented with acute coronary syndrome (ACS), of which 40% were ST elevated myocardial infarction (STEMI). 4% of the cohort had cardiogenic shock. Most PCI procedures were classified as urgent or rescue (77%) and 26% were multi-lesion procedures.

**Table 1:** Baseline characteristics according to the multimorbidity clusters

	A (n = 5912, 42%)	B (n = 853, 6%)	C (n = 553, 4%)	D (n = 6707, 48%)	Total (n = 14,025)
Age, years, mean ± SD	65.1 ± 11.3	71.8 ± 12.0	71.1 ± 10.8	60.1 ± 11.8	63.3 ± 12.1
Male, n (%)	4121 (69.7)	542 (63.5)	340 (61.5)	5402 (80.5)	10,405 (74.2)
Multimorbidity, n (%)					
Hypertension	5782 (97.9)	624 (73.3)	520 (94.4)	1308 (19.5)	8234 (58.8)
Dyslipidaemia	5414 (91.8)	12 (1.4)	482 (87.5)	2419 (36.1)	8327 (59.5)
Diabetes	1868 (31.6)	62 (7.3)	406 (73.4)	472 (7.0)	2808 (20.0)
Cerebrovascular disease	411 (7.0)	69 (8.1)	167 (30.3)	29 (0.4)	676 (4.8)
PVD	241 (4.1)	46 (5.4)	199 (36.1)	57 (0.9)	543 (3.9)
Reduced EF	831 (14.1)	442 (51.8)	311 (56.2)	1274 (19.0)	2858 (20.4)
Reduced eGFR	1143 (19.3)	756 (88.6%)	469 (84.8)	466 (6.9)	2834 (20.2)
Chronic lung disease	600 (10.2)	154 (18.1)	124 (22.4)	452 (6.7)	1347 (9.6)
OSA	292 (4.9)	2 (0.2)	51 (9.2)	91 (1.4)	436 (3.1)
Number of multimorbidity, mean ± SD	3 ± 1	3 ± 1	5 ± 1	1 ± 1	2 ± 1
Admission status, n (%)					
Elective	1910 (32.3)	106 (12.4)	127 (23.0)	1042 (15.5)	3181 (22.7)
Urgent/rescue	4002 (67.7)	747 (87.6)	426 (77.0)	5665 (84.5)	10,840 (77.3)
Cardiac status at admission, n (%)					
ACS	4044 (68.5)	749 (88.0)	427 (77.2)	5699 (85.0)	10,919 (77.9)
STEMI	1650 (27.9)	523 (61.3)	222 (40.1)	(3277 (48.9)	5671 (40.4)
Cardiogenic shock	124 (2.1)	146 (17.1)	74 (13.4)	228 (3.4)	572 (4.1)
Multi-lesion PCI procedure, n (%)	1594 (27.0)	264 (30.9)	191 (34.5)	1569 (23.4)	3618 (25.8)

SD: Standard Deviation; STEMI: ST elevated myocardial infarction; ACS: Acute coronary syndrome; PCI: percutaneous coronary intervention, PVD: peripheral vascular disease, EF: ejection fraction, eGFR: estimated glomerular filtration rate, OSA: obstructive sleep apnoea

Missing records were minimal except for records of EF (10.4%) and New York Heart Association (NYHA) class (12.8%) as presented in Supplementary Table 1. The prevalence of comorbid conditions remained stable over time, except for a decrease in dyslipidaemia and impaired EF and a slight increase in CLD in more recent years (Supplementary Table 2).

### Comorbidity clusters identified by LCA and characteristics of patients among these clusters

**Fig. 1:**
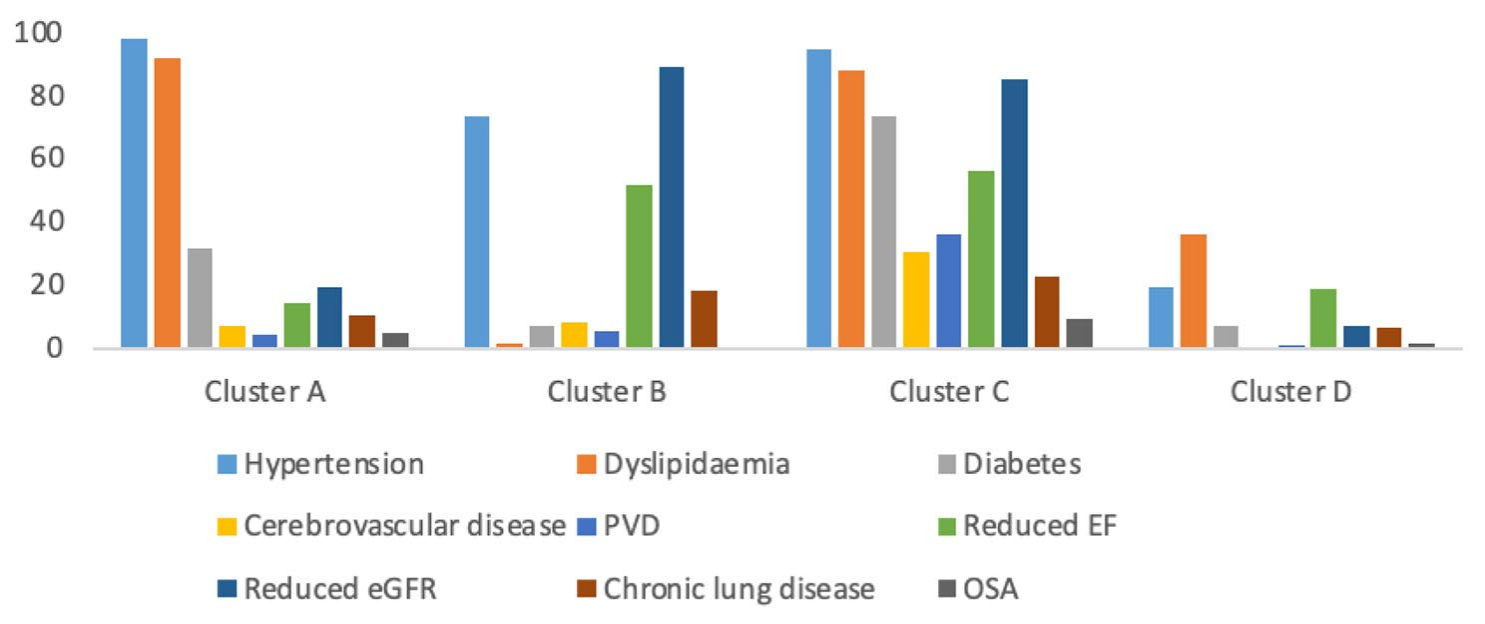
Probabilistic distribution of multimorbidity conditions within each comorbidity cluster. PVD: peripheral vascular disease, EF: ejection fraction, eGFR: estimated glomerular filtration rate, OSA: obstructive sleep apnoea

As shown in Table 1; Fig. 1, the LCA analysis identified four comorbidity clusters: [1] **cluster A** with a high prevalence of hypertension (98%), dyslipidaemia (92%) and less so diabetes (32%), [2] **cluster B** with a high prevalence of reduced eGFR (92%), hypertension (73%) and reduced EF (57%); [3] **cluster C** with a high prevalence of hypertension (94%), dyslipidaemia (88%), reduced eGFR(87%), diabetes (73%) and reduced EF (65%), [4] **cluster D** with prevalence of conditions below 20% with the exception of dyslipidaemia (36%). Each group accounted for 42%, 6%, 4% and 48% of the included population respectively. Patients in cluster B and C were likely to be older (mean age 72 ± 12 and 71 ± 11 respectively) and had a substantial high proportion of ACS, STEMI and particularly > 10% with cardiogenic shock.

### Association between identified comorbidity clusters and the outcomes

After PCI, the average, hospital stay was four days; 7% (993 events) experienced MACCE after 30 days and 13% (1857 events) at 12 months. In Table 2, compared to cluster A (as the reference group), odds ratios (ORs) of having longer length of hospital stay was statistically higher in cluster D, B and C with adjusted OR 1.03 (95%CI 1.01–1.05), OR 1.32 (95%CI 1.27–1.36) and OR 1.71 (95%CI 1.64–1.78) respectively. Patients in cluster B and D had at least twice odds of 30-day MACCE events than those in cluster A; being 2.27 (95%CI 1.73–2.96) and 2.01 (1.43–2.82), respectively. Similar pattern was observed for 12-month MACCE, and patients in cluster D had a statistically lower odds of having a 12-month MACCE compared to those in cluster A (OR 0.86, 95%CI 0.75–0.98).

**Table 2:** Association between the multimorbidity clusters and cardiovascular outcomes

	A (n = 5912, 42%)	B (n = 853, 6%)	C (n = 553, 4%)	D (n = 6707, 48%)
30-day MACCE				
Events, n (%)	330 (5.6)	188 (22.0)	107 (19.4)	368 (5.5)
Adjusted OR (95% CI)	1.00 (Ref)	**2.27 (1.73–2.96)**	**2.01 (1.43–2.82)**	0.94 (0.78–1.14)
12-month MACCE				
Events, n (%)	727 (12.3)	263 (30.8)	178 (32.2)	689 (10.3)
Adjusted OR (95% CI)	1.00 (Ref)	**1.85 (1.50–2.28)**	**2.22 (1.73–2.84)**	**0.86 (0.75–0.98)**
Length of hospital stay				
Mean ± SD	3.6 ± 4.4	6.4 ± 9.6	7.7 ± 9.9	4.0 ± 4.1
Adjusted OR (95% CI)*	1.00 (Ref)	**1.32 (1.27–1.36)**	**1.71 (1.64–1.78)**	**1.03 (1.01–1.05)**

*: Negative binomial regression IRR is reported; MACCE: major adverse cardiac and cerebrovascular events, OR: Odd ratio, CI: confidence interval. The models were adjusted for age, gender and other risk factor

## Discussion

In this study, over two-thirds of included patients had multimorbidity as defined, with the most prevalent conditions being hypertension and dyslipidaemia. Four distinctive multimorbidity clusters were associated with significant high risk of 30-day and 12-month MACCE and length of hospital stay.

Clusters B and C shared high proportions of reduced eGFR, reduced EF and hypertension but with a low proportion of diabetes in cluster B (7%). Yet, the study by Hall et al [12] using the similar LCA analysis identified only three multimorbidity clusters: [1] the high risk class with high proportion of heart failure, PVD and hypertension, [2] the medium risk class with PVD and hypertension and [3] the low risk class with low levels of interested multimorbidity with less than 5% except for high prevalence of PVD of over 20%. Although morbidities were similar in both studies the differences may be explained by how the comorbidity of interest was defined in two studies. The Hall et al. study looked at a diagnosis of chronic renal failure (defined as serum creatinine level above 2.26 mg/dl) or chronic heart failure whereas our study collected data of reduced eGFR < 60 ml/min/1.73m2 and reduced EF < 45%. Also, the Hall et al. study restricted to patients with ACS only. In our study, reduced eGFR and reduced EF were likely to co- exist and related to poor cardiovascular outcomes due to synergetic effects of traditional and uremia-related risk factors [21]. Patients with impaired kidney function tend to be under treated with preventive medicines upon discharge such as aspirin and statins [22–24] due to reticence relating to safety and benefits of these preventive medicines [25, 26]. There is a need of studies to confirm the effects of additional medicines to manage competing morbidities designed to improve the outcome after PCI in these patients [27]. Also, previous studies showed a lack of referral to cardiac rehabilitation in patients with multimorbidity [28, 29] even though they obtained similar benefits to those without multimorbidity [30–32].

Our study data was based on the MIG registry that has broad comparability to all Australian PCI patients and thus the results of our study are generalisable [33]. Compared to other data sources, clinical quality registries provide reliable, consistent and clinically relevant data. This study adopted LCA that is a data-driven approach to identify groups of patients according to the probabilities of the existence/co-existence of different conditions [34]. Using this method, we are able to understand the patterns and probabilities of multimorbidity among PCI patients in real world. In addition, LCA includes procedures for handling missing values, and therefore it can be applied to incomplete data [20].However, a key limitation of this study is the limited number of conditions recorded in the MIG registry. We did not have information on a number of relevant conditions, namely, anaemia, mental disorders, musculoskeletal disorders and obesity. Thus, our study is likely to have underestimated the association between multimorbidity and cardiac outcomes among PCI patients. Nevertheless, the nine morbidity conditions recorded in the MIG registry and thus included in the analysis are the most relevant ones to the prevention and progression of CHD. Another limitation was that the quality and accuracy of data collection determined the interval validity of the analysis. The recording of dyslipidaemia in the dataset was based on patients’ prior diagnosis of dyslipidaemia or use of statins. Therefore, it did not reflect the lipid levels at admission. Yet, the number/proportion of missing records were low, especially the recording of multimorbidity. Thirdly, our study included historical data from 2005 to 2015, during which there may have been changes in clinical practice and patient’s presentations for PCI. However, we found no clinically significant differences over time with regard to patient’s demographic characteristics nor the prevalence of the nine conditions (Supplementary file).

## Conclusion

The combination of hypertension and dyslipidaemia is prevalent in at least four in ten patients undergoing coronary angioplasty. This study showed that clusters of patients with multimorbidity had significantly different risk of 30-day and 12-month MACCE after PCI. This suggests the necessity for treatment approaches that are more personalised and customised to enhance patient outcomes and the quality of care delivered to patients in various comorbidity clusters. These results should be validated in a prospective cohort and to evaluate the potential impacts of these clusters on the prevention of MACCE after PCI.

### Electronic supplementary material

Below is the link to the electronic supplementary material.

Supplementary Material 1

## Data Availability

The datasets used and/or analysed during the current study are available from the corresponding author on reasonable request.

## List of abbreviations

PCI: Percutaneous coronary intervention
MACCE: Major adverse cardiac and cerebrovascular events
MIG: Melbourne Interventional Group
CI: Confidence interval
AMI: Acute myocardial infarction
LCA: Latent class analysis
EF: Ejection fraction
CLD: Chronic lung disease
OSA: Obstructive sleep apnoea
PVD: Peripheral vascular disease
eGFR: Estimated glomerular filtration rate
AIC: Akaike Information Criterion
BIC: Bayesian Information Criterion
NYHA: New York Heart Association
OR: Odds ratios
STEMI: ST elevated myocardial infarction
ACS: Acute coronary syndrome
