# Supplementary material for: Multimorbidity impacts cardiovascular disease risk following percutaneous coronary intervention: latent class analysis of the Melbourne Interventional Group (MIG) registry
Source: BMC Cardiovasc Disord. 2024 Jan 23;24:66. doi: 10.1186/s12872-023-03636-7 (PMC10804750; doi:10.1186/s12872-023-03636-7)
Supplement: Supplementary file 1 — Supplementary Material 1 [file 12872_2023_3636_MOESM1_ESM.docx]

Supplementary materials

Table S1: Missing data

|  | **Numbers of missing records** | **Proportion of missing records** |
| --- | --- | --- |
| **Co-morbidities** |  |  |
| **Hypertension** | 13 | 0.1% |
| **Dyslipidaemia** | 35 | 0.2% |
| **Diabetes** | 10 | 0.1% |
| **Chronic Lung Disease** | 51 | 0.4% |
| **OSA** | 36 | 0.3% |
| **Reduced EF** | 1463 | 10.4% |
| **Reduced eGFR** | 508 | 3.6% |
| **PVD** | 25 | 0.2% |
| **Cerebrovascular Disease** | 24 | 0.2% |
| **Patient Characteristics** |  |  |
| **Age** | 0 | 0 |
| **Sex** | 1 | 0.0 |
| **Smoking History** | 181 | 1.3% |
| **Family History** | 392 | 2.8% |
| **Admission status** |  |  |
| **ACS** | 7 | 0 |
| **STEMI** | 0 | 0 |
| **Cardiogenic shock** | 0 | 0 |
| **Outcomes** |  |  |
| **In hospital MACE** | 0 | 0 |
| **30-day MACE** | 0 | 0 |
| **30-day MACCE** | 0 | 0 |
| **12-month MACE** | 0 | 0 |
| **12-month MACCE** | 0 | 0 |

STEMI: ST elevated myocardial infarction; ACS: Acute coronary syndrome; PCI: percutaneous coronary intervention, PVD: peripheral vascular disease, EF: ejection fraction, eGFR: estimated glomerular filtration rate, OSA: obstructive sleep apnoea. MACCE: major adverse cardiac and cerebrovascular events.

Table S2: Characteristics of patients in MIG registry over time

|  | **2005-2009 (n=6621)** | **2010-2015 (n=7404)** | **Total (n=14025)** |
| --- | --- | --- | --- |
|  | **Demographic characteristics** | | |
| **Sex** Males | 4899 (74.0) | 5506 (74.4) | 10405 (74.2) |
| **Age [mean (SD)]** | 63.2 (12.1) | 63.5 (12.1) | 63.3 (12.1) |
|  | **Pre-existing morbidity conditions** | | |
| **Hypertension** | 3821 (57.7) | 4413 (59.6) | 8234 (58.7) |
| **Dyslipidaemia** | ***4230 (63.9)*** | ***4097 (55.3)*** | 8327 (59.4) |
| **Diabetes** | 1316 (19.9) | 1492 (20.2) | 2808 (20.0) |
| **Chronic Lung Disease** | ***570 (8.6)*** | ***777 (10.5)*** | 1347 (9.6) |
| **OSA** | 207 (3.1) | 229 (3.1) | 436 (3.1) |
| **Reduced EF** |  |  |  |
| 30-45% | ***1358 (20.5)*** | ***1293 (17.5)*** | 2651 (18.9) |
| <30% | ***110 (1.7)*** | ***97 (1.3)*** | 207 (1.5) |
| **Reduced eGFR** |  |  |  |
| eGFR (30-60 ml/min/1.73m^2^) | 1202 (18.2) | 1320 (17.8) | 2522 (18.0) |
| eGFR (<30 ml/min/1.73m^2^) | 152 (2.3) | 160 (2.2) | 312 (2.2) |
| **PVD** | 278 (4.2) | 265 (3.6) | 543 (3.9) |
| **Cerebrovascular Disease** | 343 (5.2) | 333 (4.5) | 676 (4.8) |

PVD: peripheral vascular disease, EF: ejection fraction, eGFR: estimated glomerular filtration rate, OSA: obstructive sleep apnoea

Table S3: LCA model fit test and model selection

| classes | G^2^ | AIC | BIC | CAIC | Adjusted BIC | Entropy |
| --- | --- | --- | --- | --- | --- | --- |
| 2 | 1992.33 | 2042.33 | 2231.05 | 2256.05 | 2151.6 | 0.47 |
| 3 | 992.37 | 1062.37 | 1326.57 | 1361.57 | 1215.35 | 0.49 |
| *4* | ***808.88*** | ***902.88*** | ***1257.66*** | ***1304.66*** | ***1108.30*** | ***0.54*** |
| 5 | 754.07 | 872.07 | 1317.44 | 1376.44 | 1129.94 | 0.48 |
